# Effects of Amorphous Silica Nanopowders on the Avoidance Behavior of Five Soil Species—A Screening Study

**DOI:** 10.3390/nano10030402

**Published:** 2020-02-25

**Authors:** Joana Santos, Ângela Barreto, João Nogueira, Ana Luísa Daniel-da-Silva, Tito Trindade, Mónica J. B. Amorim, Vera L. Maria

**Affiliations:** 1 Department of Biology & CESAM, University of Aveiro, 3810-193 Aveiro, Portugal; joanasilvasantos@ua.pt (J.S.); abarreto@ua.pt (Â.B.); mjamorim@ua.pt (M.J.B.A.); 2 Department of Chemistry & CICECO, University of Aveiro, 3810-193 Aveiro, Portugal; jh.nogueira@ua.pt (J.N.); ana.luisa@ua.pt (A.L.D.-d.-S.); tito@ua.pt (T.T.)

**Keywords:** engineered nanomaterials, nanosilica, terrestrial invertebrates, avoidance assay, ecotoxicology

## Abstract

Silica nanoparticles (SiO_2_NPs) are one of the most used in commercial products and biomedical tools, however, their environmental effects have not been fully described. Although negative effects of SiO_2_NPs on the behavior of freshwater invertebrates have been reported, the knowledge is limited, especially the effect of nanopowders in terrestrial organisms. Accordingly, the aim of the present study is to understand the effects of SiO_2_NPs on the avoidance behavior of five soil species, whose niche may differ thus contributing to differential harmful SiO_2_NPs effects. Hence, avoidance assays testing SiO_2_NPs concentrations of 0, 10, 100, 250, 500 and 1000 mg/kg were performed with *Enchytraeus crypticus*, *Folsomia candida*, *Tenebrio molitor*, *Porcellionides pruinosus* and *Eisenia fetida*. SiO_2_NPs induced different behavioral effects, depending on the invertebrate ecology/habitat, exposure route and physiology. *T. molitor*, *P. pruinosus* and *F. candida* did not avoid contaminated soil; however, *E. crypticus* and *E. fetida* significantly avoided SiO_2_NPs spiked soil. Since these terrestrial worms (oligochaetes) live mostly burrowed in the soil, this can provide greater opportunity for SiO_2_NPs’ uptake. On the other hand, the other tested organisms mainly living on the upper part of the soil did not avoid the SiO_2_NPs spiked soil. The avoidance data obtained here also highlight the need for further studies to understand whether (or not) the detected behavioral responses are linked to either neurotransmission processes or sensorial aspects of the biological models.

## 1. Introduction

Silicon (mostly as SiO_2_) is (naturally) highly occurring in the earth’s crust (e.g., soils) in the form of crystalline, poorly crystalline and amorphous mineral phases within a wide range of particle sizes [1]. In terms of manufacture processes, SiO_2_NPs are among the top five nanomaterials in commercial products. These nanomaterials, namely in a powdered form, have found relevant properties that make SiO_2_NPs useful in chemical, biotechnological and plastic manufacturing industries, electronics, biomedicine and agriculture [2,3,4]. However, the large-scale production of SiO_2_NPs have raised also concerns about their potential adverse effects, namely due to accumulation in specific environments [5]. Indeed, with the increasing use of SiO_2_NPs, its release into the environment may occur either from localized sources, such as industrial plants or landfills, or from non-localized sources, e.g., storm-water runoffs, runoffs from soils and from the degradation of products containing these nanomaterials (for example: personal care products, plastics, rubbers, coatings and ceramics) [6]. The addition of manufactured nanosilica can change the soil mineral composition, being frequently applied in the agriculture sector to increase seed viability and germination rate, as an ingredient in pesticides/fertilizer elements and to enhance plant tolerance to abiotic stresses [7,8,9]. Hence, it is expected that soil will be an important reservoir of engineered SiO_2_ nanopowders in variable states of aggregation and combination, making crucial a correct assessment of potential impacts in terrestrial environments and in the overall soil ecosystem [7]. These are important interdisciplinary approaches, namely because reported studies on engineered nanoparticles, have also shown specific abiotic properties (e.g., size, shape, surface-to-volume ratio and silanol group concentration) that modify toxic effects in organisms as compared to bulk silica powders [9].

Previous studies have investigated the effects of SiO_2_NPs in different soil and aquatic organisms [4,10,11,12], however, the knowledge about the impact of SiO_2_NPs exposure is still limited, namely on soil organisms. Moreover, contradictory results have been reported regarding the effects of SiO_2_NPs on survival; some studies reported no effects on *Enchytraeus crypticus*, *Caenorhabditis elegans*, *Daphnia magna* and *Danio rerio* [4,11,12], while others reported decreased survival on *D. magna* and *D. rerio* [13,14]. Regarding the effects of SiO_2_NPs on the behavior of the organisms, the few studies available mostly agree that these nanomaterials have adverse effects in this endpoint, by altering the locomotion pattern and reducing the speed of movements of *C. elegans* [15]. Color preference, photomotor and locomotor activities were also affected in *D. rerio* [3,4,13].

Soil organisms have been used as valuable ecotoxicological models for avoidance behavior assessment [16,17], since species may respond differently to chemical stimulus due to their dissimilar sensitivities [18,19,20]. All species selected for the present study are widely used in ecotoxicity evaluation due to their sensitivity to changes on soil chemical composition, easiness of cultivation in the laboratory, and their key role in the ecosystem. *Eisenia fetida* was selected because they are common in a wide range of soils, and earthworms represent 60–80% of the total soil animal/invertebrate biomass [18]. *Enchytraeus crypticus* are important members of the soil biocenosis contributing to vital processes of this environmental compartment [21]. Similarly, *Folsomia candida* are present worldwide and have an important role in soil, being involved in respiration and decomposition processes [22]. *Porcellionides pruinosus* are widely distributed and important macrodecomposers, mainly in leaf litter decomposition [23]. The *Tenebrio molitor* larva was selected as a representative of soil-dwelling organisms that feed on all types of organic matter and are restricted mainly to the upper part of the soil [24]. To the best of our knowledge, with the exception of *Tenebrio molitor*, all the species used in the present study were already successfully used in avoidance assays [18,19,25]. Nevertheless, there are no studies comparing the avoidance responses between all the species that were tested in this research.

Avoidance assays provide a rapid and valuable ecotoxicological information about contaminated soils [16,17]. Using these tests, the behavioral effects of nanoparticles such as titanium silicon oxide (TiSiO_4_), cerium dioxide (CeO_2_) and silver (Ag) have been demonstrated [6,26,27]. The assays rely on the organisms’ ability to actively avoid the contaminated soil by detecting a chemical, using chemoreceptor organs and altering their behavior [20,21]. Such a response is considered a highly relevant ecological endpoint, because if the organisms avoid a contaminated soil, the services that they provide will be compromised and the habitat function decreases, negatively impacting on the soil ecosystem and animal communities [6,22,27]. On the other hand, the escape of the organisms to “non-contaminated” soils may allow their survival, continuing their ecological performance. Besides, avoidance behavior may affect the energy budget of the organisms and change their movements, which may interfere with the soil structure [20,28]. Although the few studies available have shown pernicious consequences of SiO_2_NPs on behavior, there are no reports on the avoidance behavior of soil organisms after the exposure to these materials. Thus, this study aims to assess the effects of SiO_2_ nanopowders on the avoidance behavior of five soil species, *E. crypticus, F. candida, P. pruinosus, E. fetida* and *T. molitor,* as well as to compare their sensitivities to this material.

## 2. Material and Methods

### 2.1. Test Organisms

*Enchytraeus crypticus* were obtained from a laboratory culture kept in agar, consisting of the Bacti-Agar medium (Agar No. 1, Lab M Limited, Lancashire, UK) and a sterilized mixture of four different salt solutions: 2 mM  CaCl_2_·2H_2_O (Purity: 99.0–105.0%; Panreac, Darmstad, Germany; AppliChem, Barcelona, Spain); 1 mM  MgSO_4_ (Purity: 99.0–105.0%; Panreac, Darmstad, Germany; AppliChem, Barcelona, Spain); 0.08 mM KCl (Purity: 99.5–101.0%; VWR Leuven, Belgium) and 0.75 mM  NaHCO_3_ (Purity: 99.0–105.0%; Panreac, Darmstad, Germany; AppliChem, Barcelona, Spain), at a temperature of 19 ± 1  °C with a 16 h:8 h light:dark photoperiod. Cultures were fed on ground-autoclaved oats twice per week. Unsynchronized adults with a well-developed clitellum were used for the tests.

*Folsomia candida* were obtained from a laboratory culture kept on a moist substrate of plaster of Paris and activated charcoal (8:1 ratio), at a temperature of 20 ± 1 °C, in constant darkness. Food consisted of dried baker’s yeast (*Saccharomyces cerevisae*) provided weekly. Age-synchronized juveniles (10–12 days) were used for the test.

*Porcellionides pruinosus* were obtained from a culture of organisms hand-collected in a horse manure heap and maintained in laboratory on soil adjusted to a moisture content of 60% of soil water holding capacity (WHC), at a temperature of 20 ± 1 °C and under a photoperiod of 16 h:8 h (light:dark). Cultures were fed ad libitum with alder leaves (*Alnus glutinosa*). Only adult animals (15–25 mg wet weight) were used in these assays, excluding molting animals, those with any visible problem and pregnant females. Gender differentiation was not considered.

*Eisenia fetida* were obtained from a laboratory culture kept in opaque 24 L plastic containers, with a mixture of soil potting mix and peat adjusted at 70% of its WHC and pH between 6 and 7, and maintained at 20 ± 1 °C with a photoperiod of 16 h:8 h (light:dark). The earthworms were fed with previously frozen horse manure to kill fly eggs, if present. It was gradually thawed afterwards and used weekly as a food source, by covering the surface of the container with a 3–4 cm layer. Adult individuals were three months old, with developed clitellum and in a range of 300–600 mg of body weight.

*Tenebrio molitor* larvae were obtained from a laboratory culture kept in plastic containers with lids perforated with air holes and maintained at 20 ± 1 °C with a photoperiod of 16 h:8 h (light:dark). Cultures were fed ad libitum with oats and maintained with bran medium.

### 2.2. Test Materials

Commercial SiO_2_NPs (white nanopowder, size of 12 nm (diameter), specific surface area of 175–225 m^2^/g, purity of 99.8% trace metals basis and a water content of ≤1.50% according to the supplier) were purchased from Sigma–Aldrich (ref. 718483, St. Louis, MO, USA). According to the manufacturer information, the purchased nanoparticles are free of any organic stabilizer. This information was checked by performing the elemental analysis for C content and acquiring the infrared spectrum. The elemental analysis was carried out on a Leco Truspec-Micro CHNS 630-200-200 equipment (LabX, Midland, ON, Canada). The Fourier transform infrared (FTIR) spectrum of the SiO_2_ NPs was collected using a Bruker Optics Tensor 27 spectrometer (Bruker, Billerica, MA, USA) coupled to a horizontal attenuated total reflectance (ATR) cell, using 256 scans at a resolution of 4 cm^−1^. The structure of SiO_2_NPs was analyzed by X-ray diffraction (XRD) using a Rigaku Geigerflex Dmax-C diffractometer (Rigaku, Tokyo, Japan) equipped with a CuKα monochromatic radiation source with a step size of 0.026° and time per step of 350 s. Furthermore the specific surface area of the SiO_2_NPs was assessed by N_2_ adsorption isotherm measurements performed with a GeminiV2.0 Micromeritics instruments (Micromeritics, Norcross, GA, USA) at −196 °C. The specific surface area was determined using the Brunauer–Emmett–Teller (BET) equation for relative pressures (p/p_0_) up to 0.3 [29]. Prior to BET measurements, the samples were degassed at 120 °C under nitrogen flow overnight.

The hydrodynamic diameter and the surface charge of the nanoparticles were measured in ultrapure water through dynamic light scattering and zeta potential measurements respectively (DLS and ZP; Zetasizer Nano ZS, Malvern Instruments, Malvern, UK), at the concentrations used in the avoidance tests. Additionally, the particle size and morphology of SiO_2_NPs were also assessed by scanning and transmission electron microscopy (SEM/TEM) using a Hitachi HD-2700 microscope (Hitachi, Tokyo, Japan) operating at 200 kV. Samples for electron microscopy analysis were prepared by evaporating particle suspensions on a copper grid coated with an amorphous carbon film.

### 2.3. Test Soil and Spiking

Natural standard soil LUFA 2.2 (Speyer, Rhineland-Palatinate, Germany) was used. The main characteristics of the soil were: grain size distribution of 7.2% clay, 8% silt and 77.5% sand, pH (CaCl_2_) = 5.5, WHC of 45 g/100 g, a cation exchange capacity of 10 meq/100 g, and an organic carbon content of 1.77%. The LUFA 2.2 soil was analyzed by inductively coupled plasma optical emission spectrometry (ICP-OES; Horiba Jobin Yvon, Activa M model, Kyoto, Japan) to quantify the amount of Si. Briefly, 200 mg of soil samples were rigorously weighed. The samples were digested with 3 mL hydrochloric acid (HCl) + 1 mL nitric acid (HNO_3_) + 1 mL hydrofluoric acid (HF), using a microwave oven system. After digestion, MilliQ water was added to the samples to a final volume of 250 mL. To validate the digestion and analysis process, a certified reference material, BCR 143R, was used. The obtained percentage of recovery of this material was 105%. Additionally, the soil was characterized by XRD using a Rigaku Geigerflex Dmax-C diffractometer equipped with a CuKα monochromatic radiation source with a step size of 0.026° and time per step of 350 s.

The soil was dried (48 h, 60 °C) before use. The control soil was prepared by adding deionized water to adjust to the adequate moisture content (50% of the WHC). The nominal concentrations used in all the avoidance assays were 0, 10, 100, 250, 500 and 1000 mg SiO_2_NPs/kg soil dry weight (DW). To obtain the final concentration range, SiO_2_NPs powder was incorporated in dry soil batches and homogeneously mixed. Soil moisture was posteriorly adjusted to 50% of the WHC. Despite the tested amounts of SiO_2_ in the soil are likely higher than the predicted environmental concentrations (PECs) [10], the selection of SiO_2_NPs concentrations was based on the data available for natural silica occurrence and reported toxicological effects [3,10,30]. The literature reports amorphous silica levels in the soil between 1 and 30 g/kg [30] and also the behavioral responses (*Danio rerio*) for 300 and 1000 mg SiO_2_NPs/L [3]. The concentrations selected for the present study are non-lethal for *E. crypticus* [10].

### 2.4. Avoidance Assays

**Figure 1:**
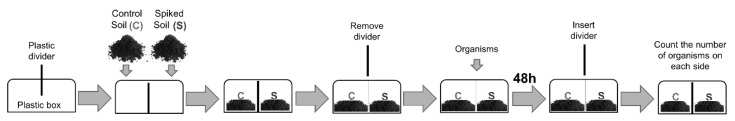
Schematic representation of the avoidance assays performed.

Assays were conducted in test containers covered with lids (containing small holes) and kept for 48 h, at 20 ± 1 °C and a photoperiod of 16 h:8 h (light:dark). As a test validation, a dual control test was performed with both compartments filled with control soil. Five biological replicates with different pools of organisms per treatment were done. The avoidance assay scheme is indicated in Figure 1.

In particular, for *E. crypticus*, the avoidance test was performed following the earthworm avoidance test guideline [16] with some adaptations as described on previous studies [28,31]. In brief, half of each container was filled with 25 g of control soil and the other half with 25 g of spiked soil. Ten adult organisms were used per replicate. At the end of the test, each side of the container was independently searched for worms.

For *F. candida*, the avoidance test guideline ISO 17512-2 [17] was followed, using the 2 chamber option [32]. Half of each plastic box was filled with 30 g of the control soil and the other half with 30 g of spiked soil. Twenty juveniles (10–12 days old) were used per replicate. After 48 h, the soil from each half of the container was separated and put into new vessels, flooded with water and the number of floating individuals was counted directly.

For *P. pruinosus*, the avoidance test was adapted from previous studies [18,23,26]. Containers were divided in two compartments, one compartment was filled with 25 g of control soil and the other with 25 g of spiked soil. Six isopods were used per replicate. At the end of the test, organisms on each side were counted.

For *E. fetida*, the avoidance test was performed following the earthworm avoidance test guideline [16]. Containers were divided into two uniform compartments, one side contained 300 g of control soil and the other side contained 300 g of spiked soil. Ten adult earthworms were used per replicate. After 48 h, earthworms in each side of the container were recorded.

For *T. molitor*, the avoidance test was performed following the earthworm avoidance test guideline [16] with some adaptations taking into account the characteristics of the species used. Containers were divided in two equal sides, half of the container was filled with 30 g of the control soil and the other half with 30 g of spiked soil. Ten organisms were used per replicate. At the end of the test, organisms on each side were counted.

### 2.5. Statistical Analysis

The avoidance response expresses the percentage of affected worms (i.e., those that avoided the spiked soil), and was calculated following the earthworm avoidance test guideline [16]. Percentage of avoidance per treatment was calculated as ***A***:

***A*** = (***C*** − ***T***) / **N** × 100

where ***C*** is the number of organisms in control soil, ***T*** represents the number of organisms on test soil and **N** is the total number of organisms used per replicate. Positive values indicate avoidance and negative values indicate a non-response or attraction to SiO_2_NPs. Percentages of avoidance (***A***) ≥ 80% indicate limited habitat function [16].

Graphics and statistical analysis were performed using the Sigma Plot 12.5 software package (Systat Software Inc., Germay). The Shapiro–Wilk and Levene’s tests were done to assess the normality and homoscedasticity of data, respectively. One-way analysis of variance (ANOVA) followed by Dunnett’s comparison post hoc test was used to assess differences between control and treatments. When data failed the normality and homoscedasticity tests, a non-parametric Kruskal–Wallis’ test was performed. All statistical analyses were performed with a significance level of 0.05.

## 3. Results

### 3.1. Characterization of SiO_2_NPs

**Figure 2:**
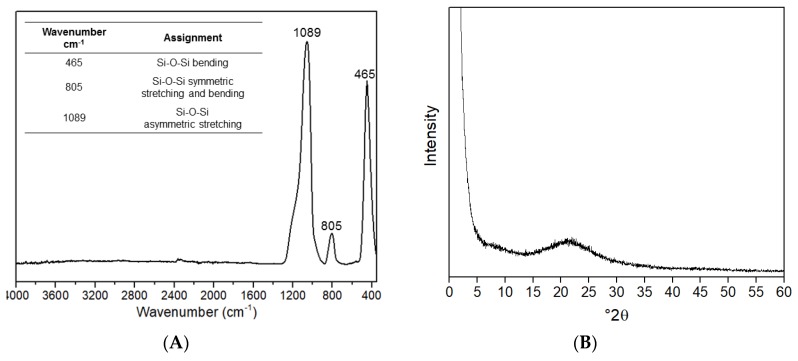
(**A**) Attenuated total reflectance (ATR)-FTIR spectrum and (**B**) X-ray diffraction (XRD) pattern of the commercial silica nanoparticles (SiO_2_NPs).

The elemental analysis of the SiO_2_NPs has revealed a carbon content of ca 0.3 wt%, which allowed us to exclude the presence of significant amounts of organic compounds, such as stabilizers, and is in agreement with the information provided by the supplier. This was further confirmed by the infrared spectroscopy analysis that did not show vibrational bands that could be assigned to organic compounds. The ATR-FTIR spectrum of commercial NPs (Figure 2A) showed the typical vibrational bands of amorphous SiO_2_ namely a broad band centered at 1089 cm^−1^ assigned to the Si–O–Si asymmetric stretching and the bands at 465 and 8055 cm^−1^, ascribed to the Si–O–Si bending and to both Si–O–Si symmetric stretching and bending vibrations, respectively [33,34,35].

As expected, the XRD data (Figure 2B) showed only a broad band, without sharp Bragg diffraction peaks that could be ascribed to crystalline phases, thus confirming the amorphous nature of the commercial silica. The BET specific surface area determined was 209.6 m^2^/g, a value that complies with the supplier product specifications.

**Figure 3:**
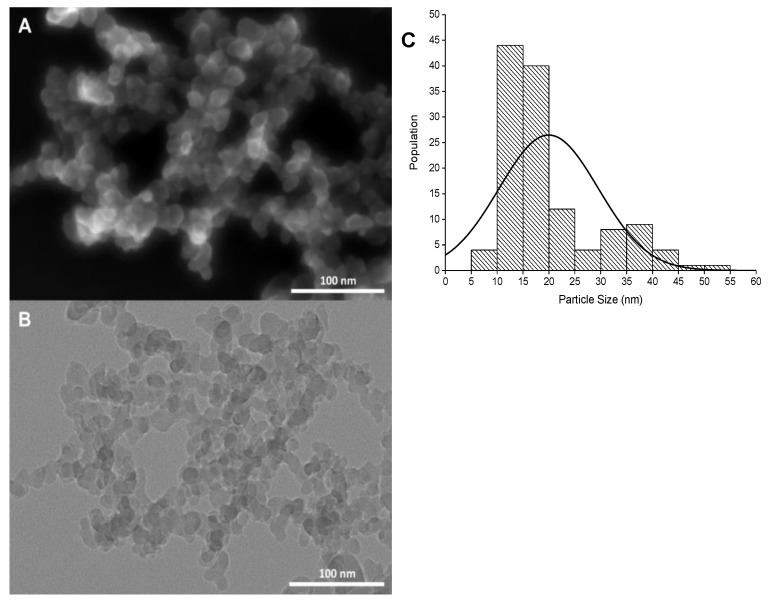
(**A**) Scanning and (**B**) Transmission Electron Micrographs (SEM and TEM) of commercial silica nanoparticles (SiO_2_NPs). Size distribution histogram (**C**) obtained from the analysis of the micrographs.

Electron microscopy analysis (SEM and TEM) demonstrated that SiO_2_NPs were spheroidal with an average size of 15.7 ± 4.1 nm. A small population of larger particles with an average size of 35.6 ± 7.1 nm (Figure 3A,B) was also observed. The microscopy analysis of the powders showed mostly large aggregates composed of smaller SiO_2_NPs. Since these SiO_2_NPs are very small and their light scattering in the respective suspensions is very low [36], the assessment of the hydrodynamic diameter by DLS resulted in unsatisfactory data. The hydrodynamic diameter was 323.9 ± 7.6 nm (polydispersity index (PDI) of 0.372) for a concentrated suspension (1000 mg/L) and PDI> 0.6 for SiO_2_NPs concentrations below 1000 mg/L (Figure 3C). However, it is worth noting that we could not infer about the aggregation state of the nanoparticles in the wet soil based on the results of DLS measurements. The ZP values of SiO_2_NPs, in ultrapure water, were consistently negative (between −24.2 and −40.6 mV; pH: 5.3 to 5.5) comparing the tested concentrations of SiO_2_NPs (Table 1) and indicated that the surface of the NPs was negatively charged.

### 3.2. Characterization of Soil

**Figure 4:**
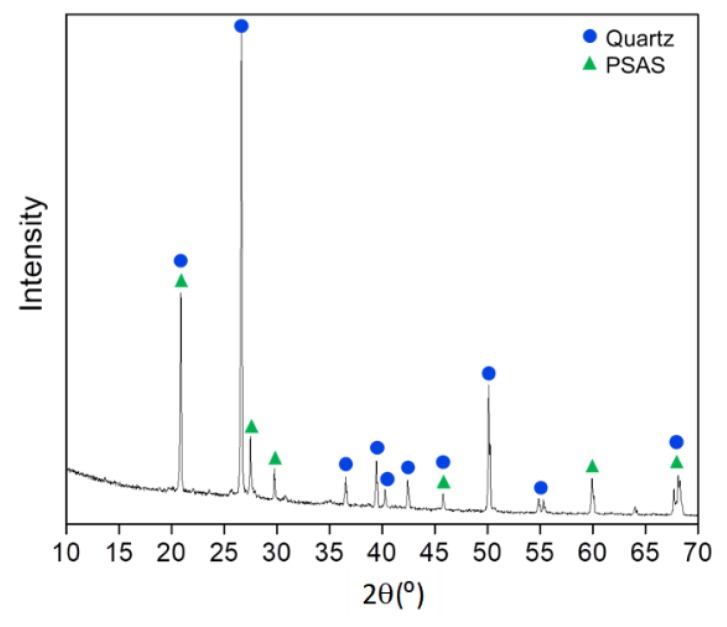
X-ray diffraction (XRD) patterns of the soil sample, with the assignment of peaks of the crystalline phases identified: quartz and Potassium Sodium Aluminum Silicate (PSAS).

The analysis by ICP-MS showed that 14 wt% of Si was present in LUFA 2.2 soil. Additionally, the powder XRD pattern of the soil showed peaks matching the diffraction pattern of crystalline SiO_2_ (quartz) [37], and potassium sodium aluminum silicate (K_0.92_Na_0.08_AlSi_3_O_8_) [38], Figure 4. The Rietveld refinement of the powder XRD data indicated the weight composition of 65/35 wt% of the components K_0.92_Na_0.08_AlSi_3_O_8_/quartz. These results indicated that the soil is naturally siliceous as it contains Si-based components in crystalline form (65% K_0.92_Na_0.08_AlSi_3_O_8_ and 35% quartz). Nevertheless, based on the collected XRD data we could not exclude the occurrence of Si in the soil samples as amorphous particulates.

There were no significant changes in soil pH (5.82 ± 0.01) within the test conditions or over the test duration (48 h).

### 3.3. Avoidance Behavior

**Figure 5:**
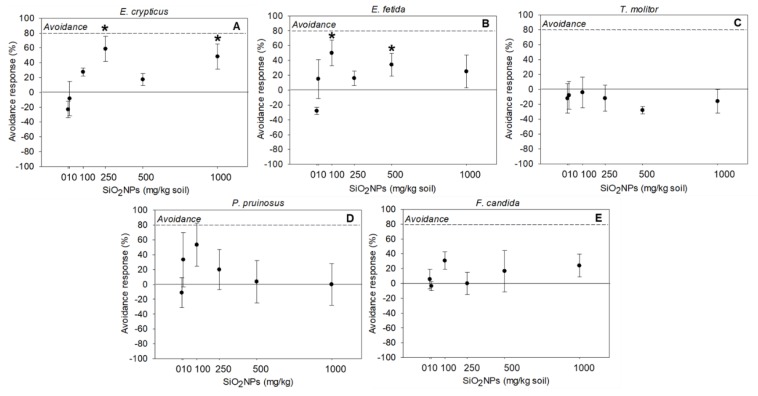
Avoidance responses of *Enchytraeus crypticus* (**A**), *Eisenia fetida* (**B**), *Tenebrio molitor* (**C**), *Porcellionides pruinosus* (**D**) and *Folsomia candida* (**E**), after 48 h, exposed to commercial silica nanoparticles (SiO_2_NPs). Results express average values ± standard errors. Dashed line indicates limited habitat function (Percentage of avoidance (***A***) ≥80%). * Significant differences to control (*p* < 0.05).

The five tested species, after 48 h, responded differently presenting dissimilar sensitivities to SiO_2_NPs exposure. *E. crypticus* and *E. fetida* showed significant (*p* < 0.05) avoidance behavior regarding SiO_2_NPs spiked soils (Figure 5A,B).

In general, there was a tendency of the organisms to avoid SiO_2_NPs spiked soils, with this response being significantly different from the control at the concentrations of 250 and 1000 mg/kg (*p* = 0.001 and *p* = 0.008, respectively) for *E. crypticus* (Figure 5A) and at 100 and 500 mg/kg (*p* = 0.0012 and *p* = 0.038, respectively) for *E. fetida* (Figure 5B). However, the calculated percentages of avoidance (*E. crypticus: A* = 58.7 and 48.3% for 100 and 500 mg/kg, respectively; *E.* fetida: *A* = 50.0 and 34.2% for 100 and 500 mg/kg, respectively) were not equal or higher than 80%, which can indicate that the habitat function will not be compromised after the exposure to the tested concentrations of SiO_2_NPs. Concerning the other tested species: *T. molitor* (Figure 5C)*, P. pruinosus* (Figure 5D) and *F. candida* (Figure 5E), the selected concentrations of SiO_2_NPs did not have a significant effect on their behavior, comparing with the control groups (*p* > 0.05). 

## 4. Discussion

In most published ecotoxicological studies, the authors have reported the characteristics of nanoparticles in stock/working solutions, namely because soil is a relatively complex medium, [39,40,41,42,43]. In the present study, the hydrodynamic size and the surface charge of the SiO_2_NPs were assessed in ultrapure water, at the concentrations used in the avoidance assays. Although this information is useful, to know the initial properties of SiO_2_NPs, not only the SiO_2_ was used here in the powdered form but their morphological characteristics also change when in contact with soil [44]. The characterization of engineered nanomaterials in real environments is crucial to understand their behavior, fate and ecotoxicity, thus helping to implement measures for their eventual use in safe conditions. Knowing the physicochemical characteristics of these materials in the receiving environmental medium (e.g., water or soil) is as important as knowing the initial properties, because they affect the fate and behavior of the nanomaterials in such media [44]. However, the assessment of the physicochemical behavior of nanomaterials in environmental matrices, especially in soils, is currently a challenge due to a lack of adequate and reliable protocols, expensive techniques and the difficulty of developing appropriate methods for better characterization [44,45]. The analysis of the electron micrographs indicated that the SiO_2_NPs have an average size of 15.7 nm, which is close to the value provided by the supplier, but also present extensive aggregation in the powders. The hydrodynamic size in ultrapure water, as assessed by DLS, was 323.9 ± 7.6 nm at the highest tested concentration (1000 mg/L). These particle dimensions clearly surpass the particle size assessed by microscopy for the primary particles, which is an indication that in the colloidal suspensions the nanoparticles remained clustered into larger structures.

After 48 h of exposure, percentages of avoidance (***A***) ≥ 80% were not recorded, which suggests that the habitat function of SiO_2_NPs contaminated soils was not compromised, even for the highest tested concentration (1000 mg/kg). Bicho et al. (2016) have reported studies that agree with this observation namely by showing no effect on the survival and reproduction of *E. crypticus* after the exposure to 100 and 1000 mg/kg of SiO_2_NPs. Although the percentages of avoidance were not large enough to represent a limited habitat function on SiO_2_NPs spiked soils, a significant change of *E. crypticus* and *E. fetida* behaviors was detected for some concentrations. This effect of SiO_2_NPs exposure should be taken into account because changes on behavior responses may affect the energy budget of the individual worms, contributing indirectly to alteration of the soil structure through induced changes in the worm movement [21]. The exposure to SiO_2_NPs (50 nm; 2.5 mg/mL) during 24 h altered *C. elegans* behavior from carving regular sinusoidal tracks into agar plates to a serrated forward locomotion pattern and reduced the movement speed [15]. The exposure to SiO_2_NPs (20, 50, 62 and 80 nm), in the concentration range 12.5–200 mg/L, induced effects on the locomotor and photomotor activities of *Danio rerio* larvae, assessed at different times of exposure, dependent on the SiO_2_NPs concentration and/or size [3]. On *Danio rerio* larvae, 25 and 50 mg/mL SiO_2_NPs induced substantial hyperactivity while 100 and 200 mg/mL elicited remarkably hypoactivity in dark periods [9]. In the same study, the total swimming distance decreased in a dose-dependent manner [9]. In addition, 168 h (seven days) of exposure to SiO_2_NPs (15 and 50 nm; 300 and 1000 µg/mL) affected the zebrafish adults learning and memory cognitive behaviors, altered their color preference and decreased their locomotive activity [3].

Soil contains crystalline silica and diverse silicates, such as quartz, plagioclase, feldspar, orthoclase and clay minerals, and a fraction of amorphous silica. This consists mainly of biogenic silica, produced by plants as phytoliths, with a variable contribution of a non-crystalline inorganic fraction, such as silica included in iron oxides/hydroxides and silica in inorganic alumino-silica coatings [46]. However, it has been described that the addition of amorphous silica to the soil affects its composition and physical properties, namely because the large surface area available promotes the sorption of water and diverse chemical species, such as phosphates and metal ions [47]. This is an important aspect because the avoidance behavior detected in *E. crypticus* and *E. fetida* may be induced by changes in the composition of chemical species in the soil due to the sorption behavior of SiO_2_NPs.

Concerning the application of avoidance assays to assess the effects of other nanomaterials, some results are already published [6,26,27]. Bouguerra et al. (2016) observed that *E. andrei* significantly avoided the soils spiked with 1000 mg/kg TiSiO_4_NPs (<50 nm). In the present study, at the same concentration, only *E. crypticus* significantly avoided the SiO_2_NPs spiked soils. Tourinho et al. (2015) found that *P. pruinosus* were able to avoid soils spiked with AgNPs (3–8 nm). At 36 mg/kg, the avoidance percentages were >80%, representing limited habitat function on the AgNPs spiked soils. In our study, the percentages of avoidance were not equal or higher than 80% for none of the tested species. Similarly, Zidar et al. (2019) reported that *Porcellio scaber* was able to detect and avoid soils spiked with AgNPs (20.4 nm) in all the selected concentrations (100, 500, 1000 and 2000 mg/kg). However, when exposed to CeO_2_NPs (<25 nm), an avoidance response of the isopods was detected for the concentration 1000 mg/kg [27]. As expected, the available results show that the effects of nanomaterials on the avoidance behavior of soil organisms depend on the type of material tested. For example, in terms of the chemical identity of different nanomaterials, the published literature shows that AgNPs induce more effects on the behavior of the terrestrial organisms than TiSiO_4_NPs and CeO_2_NPs [6,26,27].

Overall, our study showed that exposure to different concentrations of SiO_2_NPs induced different results depending on the tested species. *E. fetida* was the most sensitive species since it avoided soils spiked with 100 mg/kg, whereas *E. crypticus* only avoided soils spiked with ≥250 mg/kg of SiO_2_NPs. Kobeticová et al. (2010) reported higher sensitivity of *E. fetida* in comparison to *E. crypticus* and *Enchytraeus albidus,* after the exposure to solid industrial wastes. The different sensitivities of the organisms to a specific contaminant suggest that a set of organism’s species should be used in avoidance tests, since the responses of the organisms may intensely vary according to their ecology, physiology and the exposure route. Our study indicates that organisms mainly restricted (live) to the upper part of the soil did not avoid the SiO_2_NPs spiked soils.

Gainer et al. (2019) suggested that species traits might explain the differences in the avoidance responses of the soil invertebrates. For example, the close association of some organisms with soil pore water may explain the different avoidance responses between species because it affects the bioavailability of the chemical to the organism [20]. Collembolans are mainly exposed to soil pore water, earthworms to both pore water and soil particles by dermal and oral contact and woodlice to food (decaying leaf material), soil particles by ingestion and to a limited extent to soil pore water. The exposure route is also an important aspect to consider when comparing the responses between different species after exposure to the contaminants. Direct uptake of the contaminants via the skin is an important route of exposure in soil organisms. The negatively charged cuticle of nematodes, for example, has been shown to attract nanoparticles, to a greater degree than a comparable bulk material [48]. However, the major route of exposure may be by ingestion of contaminated soil particles or contaminated food [49]. These differences in ecology/physiology and the exposure route may explain the observed effects caused by SiO_2_NPs in the five soil species tested.

It seems that the chemoreceptors of *E. fetida* and *E. crypticus* detected the presence of SiO_2_NPs on the soil, processed this information via the nervous system and initiate a response to the stimulus translated as avoidance behavior [31]. The inability of chemoreceptors of *P. pruinosus*, *T. molitor* and *F. candida* to detect SiO_2_NPs, may explain the lack of avoidance response by these species. However, the traits/anatomy of these three species may also justify this result. They walk on top of the soil and in the porous between soil particles, hence reducing exposure compared to worms that dwell in the soil. Additionally, they have a chitin exoskeleton, which may restrict the dermal contact of the organism with the contaminant. On the other hand, the neurotoxic effects of SiO_2_NPs already reported for different organisms [3,4,15] may also explain the no escape of the organisms to non-spiked soil. As previously described, for avoidance to occur, the danger must be first perceived, which may not happen if the organism is ‘blinded’ in some capacity. The danger may be not recognized if the organism is cognitively confused or impaired [50]. A correlation between non-avoidance of *F. candida* and acetylcholinesterase inhibition was previously reported [22]. Bicho et al. (2015) showed that the non-avoidance behavior of *E. crypticus* might be related with the gamma-aminobutyric acid system. One of the main concerns are the implications of this kind of response: If organisms are not able to avoid a certain compound in the field, the effects of the contaminant on the organisms may be much higher [51,52].

Finally, the interpretation of the effects detected on the present study must be taken in consideration the possible presence and interference of other constituents that organisms may have been exposed. Although the physicochemical characterization performed by us plus the information from the supplier did not reveal unexpected chemical species, we cannot totally exclude their presence because even in vestigial amounts could contribute to the observed effects.

## 5. Conclusions

Our findings demonstrated that amorphous SiO_2_NPs lead to a change in the behavior of some soil organisms. They presented avoidance behavior to SiO_2_NPs spiked soils (250 and 1000 mg/kg for *E. crypticus* and 100 and 500 mg/kg for *E. fetida*). The detected behavior may be associated among other factors with the changes in the soil composition due to the sorption behavior of the SiO_2_NPs. A lack of avoidance response by *P. pruinosus*, *T. molitor* and *F. candida* was also found. The obtained data by this screening study highlighted the need for further studies to understand the neurotoxic effects of SiO_2_NPs to soil organisms, since the detected behavioral responses might be associated with neurotransmission processes that could be induced by nanoparticles.

## Figures and Tables

**Table 1:** Zeta potential measurements of commercial silica nanoparticles (SiO_2_NPs) in water. The results are presented as mean value ± standard deviation.

Concentration (mg/L)	pH	Zeta Potential (mV)
10	5.3	−30.2 ± 0.3
100	5.5	−40.6 ± 0.5
250	5.4	−24.2 ± 0.7
500	5.4	−30.2 ± 1.5
1000	5.4	−32.4 ± 0.1
